# “Because There’s Experts That Do That”: Lessons Learned by Health Care Organizations When Partnering with Community Organizations

**DOI:** 10.1007/s11606-023-08308-y

**Published:** 2023-07-18

**Authors:** Laura B. Beidler, Caroline Fichtenberg, Taressa K. Fraze

**Affiliations:** 1 The Dartmouth Institute for Health Policy and Clinical Practice, Geisel School of Medicine, Dartmouth College, NH Lebanon, USA; 2 Department of Family and Community Medicine, University of California, CA San Francisco, USA; 3 Social Interventions Research and Evaluation Network (SIREN), Center for Health and Community, University of California, CA San Francisco, USA; 4 Philip R. Lee Institute for Health Policy Studies, University of California, CA San Francisco, USA

## Abstract

**Background:**

Health care organizations’ partnerships with community-based organizations (CBOs) are increasingly viewed as key to improving patients’ social needs (e.g., food, housing, and economic insecurity). Despite this reliance on CBOs, little research explores the relationships that health care organizations develop with CBOs.

**Objective:**

Understand how health care organizations interact with CBOs to implement social care.

**Design:**

Thirty-three semi-structured telephone interviews collected April–July 2019.

**Participants:**

Administrators at 29 diverse health care organizations with active programming related to improving patients’ social needs. Organizations ranged from multi-state systems to single-site practices and differed in structure, size, ownership, and geography.

**Measures:**

Structure and goals of health care organizations’ relationship with CBOs.

**Results:**

Most health care organizations (26 out of 29) relied on CBOs to improve their patients’ social needs. Health care organization’s goals for social care activities drove their relationships with CBOs. First, one-way referrals to CBOs did not require formal relationships or frequent interactions with CBOs. Second, when health care organizations contracted with CBOs to deliver discrete services, leadership-level relationships were required to launch programs while staff-to-staff interactions were used to maintain programs. Third, some health care organizations engaged in community-level activities with multiple CBOs which required more expansive, ongoing leadership-level partnerships. Administrators highlighted 4 recommendations for collaborating with CBOs: (1) engage early; (2) establish shared purpose for the collaboration; (3) determine who is best suited to lead activities; and (4) avoid making assumptions about partner organizations.

**Conclusions:**

Health care organizations tailored the intensity of their relationships with CBOs based on their goals. Administrators viewed informal relationships with limited interactions between organizations sufficient for many activities. Our study offers key insights into how and when health care organizations may want to develop partnerships with CBOs.

**Supplementary Information:**

The online version contains supplementary material available at 10.1007/s11606-023-08308-y.

## Introduction

Social care—where health care organizations try to improve patients’ social conditions such as food, housing, and economic insecurity—is quickly becoming a standard care delivery practice. ^1–6^ Social care includes a broad range of activities such as screening patients for social risk factors, providing patients with referrals to local services, developing internal programs to address social needs, and partnering with community-based organizations (CBOs) ^7^. The Centers for Medicare and Medicaid Service (CMS) and the National Committee for Quality Assurance (NCQA) have accelerated health care organizations’ investments in social care by adopting social risk screening quality metrics. ^8,9^

CMS hopes that by requiring hospitals, and with outpatient providers proposed for the future, to screen patients for health-related social needs, it will spur health care organizations to develop meaningful collaborations with CBOs ^9^. NCQA will not only measure payer’s social risk screening, but also the share of members who receive an intervention (often through resource connection) to address their social needs as part of their Healthcare Effectiveness Data and Information Set ^8^. Health care organizations often rely on established, external organizations to address patient needs, such as providing tailored meals to diabetic patients, ^10^ offering transitional housing ^11^, or when they refer patients with social needs to CBOs for services. ^6,12,13^

Coordination with CBOs is a significant implementation challenge for health care organizations ^14^ because of differences between health care systems and CBOs including distinct financial models, missions, leadership structures, cultures, and priorities ^15–19^. Yet little is known about how health care organizations can overcome these barriers. To address this gap, we interviewed administrators at a diverse set of health care organizations to identify practical solutions to challenges experienced when developing relationships with CBOs. These findings offer guidance to health care organizations seeking to collaborate with CBOs as part of their approach to improve patients’ social conditions.

## Methods

### Data Collection

In April–July of 2019, we conducted 33 semi-structured interviews with administrators at 29 health care organizations. ^6,12,13,20^ All interviewed organizations had already established programs to identify and address patients’ social needs, such as food, housing, transportation, utilities, or other needs related to economic insecurity. The local Institutional Review Board approved this study.

We identified organizations using two methods. First, we randomly selected primary care practices and health care systems that responded to the National Survey of Health Care Organizations and Systems (NSHOS) and indicated they screened patients for social risks. ^4,21^ NSHOS is a suite of nationally representative surveys that were conducted in 2017–2018. ^4,21–24^ We used social risk screening as a proxy measure to identify organizations that we anticipated were engaging in other social care activities. Second, to ensure we sampled organizations engaged in a broad range of social care activities, we also conducted internet searches to identify organizations that were publicizing their social care efforts (e.g., press releases that highlighted social care efforts and news articles discussing programs).

We emailed leaders at sampled organizations and asked them to connect us with the individual at their organization who was best suited to describe the organization’s social care efforts. All interviewees were responsible for administering social care activities within their organization (we refer to these individuals as administrators). We conducted outreach in waves, adjusting each wave based on which organizations agreed to participate in the prior wave, to ensure a diverse sample. We continued data collection until we reached saturation and no longer observed new themes during interviews ^25^. In total, we contacted 64 organizations (34 from NSHOS and 30 from web searching). In total, we conducted 33 interviews with 29 organizations (11 from NSHOS and18 from web searching). At 4 organizations, we conducted a second interview to gain additional information.

Interviews focused on organization’s activities that aimed to identify and address patients’ social needs. Semi-structured interviews followed an interview guide (see the Appendix) that was designed to explore common social care activities—including those part of the Accountable Health Communities model from CMS and those described in the National Academies of Sciences, Engineering, and Medicine report on social care. ^7,26^ Specifically, the interview guide focused broadly on social risk screening, social needs referrals ^12^, case management assistance ^6^, other activities targeting patients with social needs (e.g., developing FoodRx programs), and interactions with CBOs (e.g., independent partnerships, community-based partnerships, and referral interactions). All interviews lasted approximately 60 min. Interviews were conducted via telephone, recorded, and professionally transcribed. Additional details on the study sample and interview methodology can be found in prior publications. ^6,12,13,20^

### Data Analysis

Trained qualitative researchers conducted initial coding of transcripts using a codebook that aligned with broad domains in the interview guide. All coding was conducted using QSR NVivo. ^27^ All coders conducted iterative double coding on a subset of transcripts until all coders agreed and were confident about the consistency between coders. Then, for all transcripts, one coder conducted initial coding and the first author conducted an unblinded second pass at coding. We conducted analysis-specific coding on health care organizations’ relationships with CBOs (which spanned interview guide domains) defined as “information about relationships with community partners, strategies to improve relationships, buy-in, and engagement.” The first author sub-coded all transcripts and the senior author reviewed the sub-coded data. Two authors (L.B. and T.F) met weekly to discuss coding. We analyzed coded data using an iterative approach and created a detailed memo that explored relationships between health care organizations and CBOs. ^28–30^ Our analysis approach was informed by prior literature on challenges health care organizations and CBOs may have when interacting such as differing motivations, financial structures, and resource capacities. ^5,15,18,31^ We used a matrix coding approach to examine how each organization fit within each theme. ^30,32^

## Results

Nearly all (26 of 29) interviewed administrators reported having relationships with CBOs as part of their social care activities (e.g., community-wide partnerships, targeted programs, or referrals). Interviewed organizations varied in size, structure, safety-net status, and geography with themes observed consistently across organizational characteristics (see appendix for details on interviewed sites).

Health care administrators were motivated to rely on CBOs rather than developing their own programs (e.g., operating a food pantry or building transitional housing) to improve patients’ social conditions because it was more cost effective and required fewer staffing resources. Administrators emphasized that they valued the contributions of CBOs and felt that CBOs were better suited than health care organizations to deliver social services.

### Health Care Organization’s Goals for Social Care Activities Drove Their Relationships with CBOs

Many health care organizations provided patients with a one-way social needs referral to CBOs with the assumption that the CBO could assist patients. In these cases, health care organizations used workflows that did not necessitate formal relationships with CBOs. In many referral programs, health care organizations did not regularly communicate with CBOs except when informal, peer-to-peer communication between staff at organizations was necessary (i.e., to confirm services, eligibility) (Table 1).

**Table 1:**
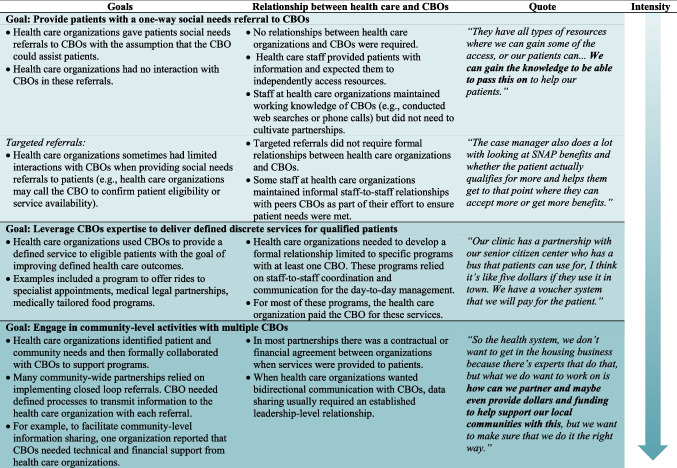
Health Care Organization’s Goals when Interacting with CBOs

In contrast, some health care organizations engaged CBOs to deliver discrete services to their patients. These narrow partnerships tended to focus on one social need with a defined scope of service (e.g., food boxes, medical-legal partnerships, or transportation services) often targeted to a defined patient population (e.g., seniors or patients with specific clinical conditions). Health care organizations were motivated to develop these types of partnerships when they identified that a specific clinical outcome (e.g., HbA1c) was commonly impacted by a social risk factor (e.g., poor nutrition due to food insecurity) among their patients.

*The unique part of the [food bank] partnership is, of course, those patients who identify with food insecurities, they also need to be a Medicaid patient in order to participate in this [food bank] piece. […] If they’re identified as [food bank] eligible,**** our care coordinator reaches out to the patient, confirms that they still have an insecurity, and then, if they say they would like to participate, then the name is given to [food bank] and then they do all of the administrative work.***

On the other end of the spectrum, several health care organizations engaged in community-level activities with multiple CBOs. These relationships usually included leadership-level communications and commitments, defined activities, and, in some instances, contractual agreements. Many administrators emphasized that they were originally motivated, in part, to engage in community-level partnerships with multiple CBOs to facilitate active bidirectional data sharing, including social needs referral platforms. While some hoped that referral platforms would be easy to implement, most found that it required significant planning and co-development of tools and processes. Some health care organizations discovered that CBOs may lack necessary infrastructure to develop robust partnerships.

### Key Collaboration Recommendations

We identified four key recommendations based on struggles described by health care organizations when developing formal partnerships with CBOs (Table 2). These recommendations are likely most useful for health care organizations that are planning to leverage CBOs’ expertise to deliver defined discrete services or those that are planning to engage in broader community-wide partnerships:

1. *Engage early*: Administrators highlighted the importance of engaging proactively with potential partners. When health care organizations did not include potential partners in initial planning, they reported setbacks due to misaligned goals as well as potential partners having neither the capacity nor interest in advancing the health care organization’s goals.
2. *Establish shared purpose for the collaboration*: Administrators stressed the importance of ensuring that participating organizations aligned organizational-level goals before embarking on new programs. Some health care organizations approached this by carefully selecting partners that they knew shared their goals while others worked with potential partners to co-develop program goals that aligned with both organizations’ overall goals.
3. *Determine who is best suited to lead*: After establishing goals for the collaboration, it was important to intentionally determine which organization should lead each part of the effort. Several organizations faced setbacks when a clear leader was not proactively selected. Even though health care administrators often have more resources than their CBO partners, the CBO may be better suited to lead efforts. When health care organizations assumed a leadership role even though the CBO was better suited to lead, health care administrators reported experiencing multiple challenges—including misaligned goals, miscommunication between organizations, and limited impact of efforts.
4. *Avoid making assumptions*: Administrators highlighted that it was vital to avoid assuming that partners had the capacity to or interest in addressing the needs they identified (e.g., discrete services to patients, significant increase in patient referrals, or other health care driven goals). Many CBOs had limited and fluctuating budgets, which influenced their capacity to address clients’ needs.

**Table 2:** Recommendations for Health Care Organizations when Developing Relationships with CBOs

Challenges	Successes
Engage early with potential partners	
Several health care organizations reported waiting too long to engage CBOs. They sometimes made key decisions, such as which referral platform to use, without input from CBOs, which created challenges to implementation “The success really hinges on whether a community partner uses its tool or not. If they won’t use it or don’t feel part of the process, then it’s just another tool that healthcare workers are trying to integrate into their workflow and it’s just not valuable[.]”	Health care organizations emphasized that including all partners from the beginning of the initiative helped ensure long-term engagement “We showed respect to our community and really went to visit them to see how they worked starting out. One of the perceptions in the community was that health systems looked down on community organizations. And we wanted to do everything we could to dispel that myth. And I think we’ve been relatively successful with it.”
Establish clear and shared goals for programs	
Partnerships were typically initiated by health care organizations who came with established goals that were not necessarily aligned with the scope or capacity of CBOs “I think that collaborating with community-based organizations is key**. I think the medical systems are often siloed from the social service systems**, so developing collaborations, I think, within communities and regions to look at this is important.”	Health care leaders engaged with CBOs to understand their priorities prior to designing programs “**Before we did anything with this plan, we pulled together a group of community, health system, and university partners to** just help us guide what it was we were going to do. As we began to look at what we were interested in, they made recommendations about who needed to be at the table.”
Determine who is best suited to lead the program and empower them	
Health care organizations, despite recognizing the expertise of CBOs, sometimes struggled to adequately cede leadership to CBOs (especially when health care organizations were providing financial support) “It’s very difficult to do a partnership if you can’t stay in sync in with each other **because one person is wanting to always take control and run it over**.”	Health care organizations should allow CBOs to lead efforts within their areas of expertise “We see it as a partnership or a collaboration where the strength that we bring is around health care and support services, and the strength that the developers bring is around housing, so we don’t run the housing piece of it. We partner with the people that do housing, **so it’s really from a strength-based perspective**. What are we good at and what are they good at?”
Avoid making assumptions about the needs or capacity of other organizations	
Some organizations noted that it was key to assess both the interest of potential partners and to understand their capabilities. For example, CBOs may not be interested in a referral platform “So we’ve already done assessments with the community-based organizations to see, okay, if we have this system in place, do you have computer technology in your organization, because a lot of these community-based organizations and social service systems are **very small and running on a shoestring, so they may not have the ability to link in.**”	Assessing CBOs’ interest in and capacity for a partnership is important to ensuring the partnership is successful “Be willing to listen and not speak. Don’t just throw money at the issue and then not be present. I think you really need to be present at the table as part of the solution. And don’t come in saying you have all the answers and that’s the way it should be done. Don’t assume that a community wants to work on a problem that you think they should work on. **They might have a completely different agenda and completely different issues that they want to focus on. I think it’s really more about listening and just being present.”**

Underlying these recommendations, as one administrator highlighted, when partnering with CBOs, health care organizations may need to change how they conceptualize and approach social care:

*I think one of the biggest problems with health care systems, especially health care providers, is that they’re very impatient. They think that because they’re used to cutting or used to giving a pill and seeing a response, and public health issues and health issues like this, social issues like this, take time, ****and relationships take time to develop****. So I think that’s the other thing, is to be patient and not expect things to happen overnight. We aren’t going to fix poverty in a year.*

## Discussion

Most health care administrators tailored the intensity of their relationships with CBOs to meet their goals and align with the structure of their social care activities. Administrators viewed informal relationships with limited interactions between organizations sufficient for many activities, such as social needs referrals. In other cases, health care organizations wanted to develop specific programs targeted at improving the health care outcomes of their socially disadvantaged patients and thus developed formal partnerships with CBOs. Finally, some health care organizations developed formal partnerships with CBOs to enhance community health.

If health care organizations’ social care efforts are based on referrals to CBOs, then limited, constrained interactions with CBOs may be sufficient. Health care organizations already face significant challenges including the care team workforce, ^33,34^ an aging and clinically complex patient population ^35,36^, and disparities in clinical outcomes ^37^; therefore, the opportunity costs for any new partnership may be steep. ^13^ Further, health care organizations and CBOs have different organizational structures, financial models, stakeholders, and areas of expertise. ^15,18^ Considering that health care organization’s mission is patient-centric and there are limited resources available for social care, it is not surprising that many interviewees did not engage in sweeping community-wide partnerships to improve social conditions. It is concerning that while limited interactions with CBOs may be sufficient to implement social needs referrals, referrals alone may not be sufficient to improve social conditions for many patients given that CBOs are often structured as short-term, acute assistance models. ^38^ This raises fundamental questions about the efficacy of a referral-based approach to social care.

Our study also offers insights for health care organizations interested in investing in deeper partnerships with CBOs. CMS hopes that quality metrics requiring hospitals, and likely outpatient providers in the future, to screen patients for social risk factors will facilitate community-level partnerships. ^9^ Yet our study suggests health care organizations may need support from CMS and others to effectively implement and scale social care activities via meaningful partnerships with CBOs. In Massachusetts, for instance, the state helped foster relationships between Medicaid accountable care organizations (ACOs) and CBOs by offering trainings on health-related social needs for the ACOs, building capacity among CBOs, support to developing data sharing agreements, and flexible funding to increase capacity. ^39^ Other states have focused on providing technical resources such as access to referral platforms. ^40,41^ One challenge is that because health care organizations are largely uncompensated for social care activities, they may aim to minimize their investments in these programs (i.e., rely on one-way referral programs). ^13^ One lesson we uncovered was that even though health care organizations felt external pressure to act quickly to improve patients’ social conditions, administrators learned the importance of being a *partner* to CBOs rather assuming leadership over programs. Initiating a partnership is a delicate process that should consider when and how a collaboration should be formed and who should be involved. Initiating relationships between health care organizations and CBOs is further complicated given health care organizations are influential due to their greater financial resources and capital. As a result, prior research has found that CBOs are adapting their processes to meet the needs of health care organizations ^31^; our study illustrates the importance of both health care organizations and CBOs adapting to meet shared goals. Another lesson was that it can be a challenging balance for health care organizations to keep in mind their own organizational mission and goals while being open to the ideas surfaced by others. If health care organizations are engaging CBOs to solicit help resolving a specific patient need, such as housing support for high-risk patients to reduce unnecessary emergency department visits or healthy access to food for patients with chronic conditions adversely affected by nutrition (i.e., diabetics), then more partnerships focused on specific needs may be appropriate.

Our study has a few key limitations. First, as a qualitative study, our results are not meant to be generalized to all health care organizations and should be used to provide context to advance research and to aid health care organizations considering how to partners with CBOs. Second, our interviews were conducted with administrators at health care organizations. While these individuals are likely the best suited to discuss organizational relationships, they may be unaware of interactions with CBOs that occur outside of their programmatic area. Third, our study was designed to ensure representation of diverse types of organizations in our interviews rather than to compare between different types of organizations. Lastly, our data is all from the perspective of health care organizations and we do not have data or insights from the CBOs who are engaging with the interviewed health care organizations (i.e., we are not able to contextualize the CBOs such as the organizational characteristics).

Policymakers and others are emphasizing the importance of meaningful community-level transformation. ^42–49^ Our study suggests that health care organizations may not view such partnerships as necessary especially if their key social care activity is referrals (which is common). If policymakers, as CMS suggests, want more meaningful partnerships, then health care may need stronger incentives and implementation supports.

### Supplementary Information

Below is the link to the electronic supplementary material.

Supplementary file1 (DOCX 29 KB)

## Data Availability

The data analyzed during the current study are available from the corresponding author on reasonable request.
