# Supplementary material for: “Because There’s Experts That Do That”: Lessons Learned by Health Care Organizations When Partnering with Community Organizations
Source: J Gen Intern Med. 2023 Jul 18;38(15):3348–54. doi: 10.1007/s11606-023-08308-y (PMC10682338; doi:10.1007/s11606-023-08308-y)
Supplement: Supplementary file 1 — Supplementary file1 (DOCX 29 KB) [file 11606_2023_8308_MOESM1_ESM.docx]

**Appendix**

Appendix Table 1: Interviewee Categories

| **Interviewee Categories** | **Description** | **Examples** |
| --- | --- | --- |
| Executive Leadership | Individuals primarily responsible for overseeing the operations of the entire organization | Chief Executive Officer, Chief Clinical Officer |
| Program Management | Individuals who oversee specific departments or services | Program Manager, Community Relations Manager, Eligibility Supervisor |
| Case Management Staff | Individuals who worked within case management teams and who focused primarily on case management activities | Nurse Care Manager, Community Health Worker, Social Worker, Navigator |
| Practicing Clinician | Individuals whose primary role was the provision of medical care | Physician |

Appendix Table 2: Organizational Characteristics

| **Organization Number** | **Description** | **Composition** | **Number of interviews** | **Interviewee(s) Role** |
| --- | --- | --- | --- | --- |
| 1 | Urban family medicine clinic in the Midwest (10 to 20 providers) | Single primary care delivery site | 1 | Program Management |
| 2 | Health system in the Northeast | Hospital, primary care and specialty delivery sites | 1 | Program Management (2) |
| 3 | Coalition of community health centers in the West | Primary care clinics | 1 | Executive Leadership, Program Management |
| 4 | Health system in the West | Hospitals, primary care and specialty delivery sites | 1 | Executive Leadership |
| 5 | Health system in the West | Hospitals, primary care and specialty delivery sites | 1 | Executive Leadership |
| 6 | Rural FQHC in an area that covers two states in the South (1 to 10 providers) | Single primary care delivery site | 1 | Executive Leadership |
| 7 | Health system in the Northeast | Hospitals, primary care and specialty delivery sites | 1 | Executive Leadership, Program Management (2) |
| 8 | Rural healthcare system that includes hospitals in two states in the Northeast | Hospitals, primary care and specialty delivery sites | 2 | Executive Leadership, Program Management/Practicing Clinician |
| 9 | Suburban FQHC with multiple clinical delivery sites in the West | Primary and specialty care delivery sites | 1 | Program Management |
| 10 | Urban FQHC with multiple locations in the West | Primary and specialty care delivery sites | 1 | Program Management |
| 11 | Accountable care organization in the Northeast | Hospitals, primary care and specialty delivery sites | 1 | Executive Leadership |
| 12 | Large health system in the Northeast that also manages its own health plan | Hospitals, primary care and specialty delivery sites; health plan | 2 | Executive Leadership, Case Management Staff |
| 13 | Small rural practice in the Northeast | Single primary care delivery site | 1 | Program Management (2), Practicing Clinician |
| 14 | Large suburban FQHC in the South | Primary care delivery sites | 1 | Executive Leadership, Program Management, Case Management Staff |
| 15 | FQHC suburban in the West | Primary and specialty care delivery sites | 1 | Program Management |
| 16 | Urban practice in the West (less than 10 providers) | Single care delivery site | 1 | Case Management Staff |
| 17 | Rural community health center in the Midwest (20 to 40 providers) | Primary care delivery sites | 1 | Case Management Staff |
| 18 | Rural community health center in the West | Primary care delivery sites | 1 | Program Management |
| 19 | Health system in the Midwest | Hospitals, primary care and specialty delivery sites | 1 | Program Management (2) |
| 20 | Urban system in the Northeast | Hospitals, primary care and specialty delivery sites | 1 | Executive Leadership, Program Management |
| 21 | Suburban community health center in the West | Primary care delivery sites | 2 | Executive Leadership, Program Management |
| 22 | Health system in the Northeast | Hospitals, primary care and specialty delivery sites | 1 | Executive Leadership |
| 23 | Health system in the Northeast | Hospitals, primary care and specialty delivery sites | 2 | Program Management |
| 24 | Health system in the Midwest | Hospitals, primary care and specialty delivery sites | 1 | Executive Leadership, Program Management (2) |
| 25 | Health system in the South | Hospitals, primary care and specialty delivery sites | 1 | Program Management |
| 26 | Urban community health center in the South | Primary care delivery sites | 1 | Executive Leadership |
| 27 | Urban FQHC in the Midwest (20 to 50 providers) | Single primary care delivery | 1 | Executive Leadership |
| 28 | Rural independent practice in the Northeast (less than 10 providers) | Single primary care delivery site | 1 | Practicing clinician |
| 29 | Urban academic health system in the South | Hospitals, primary care and specialty delivery sites | 1 | Executive Leadership, Program Management |

Appendix Table 3: Interview Guide Domains

| **Domain** | **Sub-domains** |
| --- | --- |
| Organizational Characteristics | - Organization size and structure - Interviewee role - Motivations for social needs work - Populations served - Participation in delivery reforms |
| Screening | - Which patients screened - Needs screened for - Screening workflows, methods, tools used - Staff involved with screening - Follow-up processes - Access to screening results - Frequency of screening - Reason for starting screening - Development of screening program - Engagement with clinicians - Buy-in from staff - Plans for scaling, changing screening program - Common needs patients have |
| Referrals | - Workflow - Staff involved - Tailoring to patients - Variation between locations, patients - Referral lists, referral platforms   - Development   - Maintenance   - Staff involved   - Tracking use of referrals - Role of clinicians - Buy-in from clinicians and patients - Follow-up processes - Closed loop referrals - Common challenges with referrals |
| Assistance | - Workflow - Types of assistance offered - Staff involved - Staff training - Variation between patients - Engagement with community organizations - Communication with   - Patients   - Clinicians   - Other staff - Centralized vs. decentralized programs - Tracking of patients/data collection - Common challenges - Reason for starting assistance work - Program development - Changes made to program |
| Need specific programming (e.g., food, housing, transportation) | - Internal programs - External programs - Funding - Services - Types of patients - Development processes |
| Interactions with community-based organizations (CBOs) | - Types of partners - Role of:   - Health care organization   - CBO - History of partnerships - Involvement of CBO in program development - Formalized or ad-hoc - Contractual relationships - Types of patients served - Data/records sharing |
| Overview/Reflection | - Challenges faced - Challenges solved - Overlap with care management - Organizational buy-in - Advice for other organizations - Organizational goals - Needed support (financial, resources, policy) |
